# Development and preliminary evaluation of a quality of life measure targeted at dementia caregivers

**DOI:** 10.1186/1477-7525-7-56

**Published:** 2009-06-21

**Authors:** Barbara G Vickrey, Ron D Hays, Michele L Maines, Stefanie D Vassar, Jaime Fitten, Tony Strickland

**Affiliations:** 1 University of California, Los Angeles Department of Neurology, 710 Westwood Plaza, Los Angeles, California 90095-1769, USA; 2 UCLA Department of Medicine, Division of General Internal Medicine & Health Services Research, 911 Broxton Plaza, Los Angeles, California, 90095-1736, USA; 3 UCLA Department of Psychiatry and Biobehavioral Sciences, 760 Westwood Plaza, Los Angeles, California, 90095-1759, USA

## Abstract

**Background:**

Providing care for individuals with a progressive, debilitating condition such as dementia can adversely impact the quality of life (QOL) of informal caregivers. To date, there is no existing caregiver quality of life measure for dementia caregivers with breadth of coverage or that is applicable to caregivers of diverse ethnic backgrounds. The purpose of this study was to develop and evaluate a caregiver-targeted quality-of-life measure (CGQOL) for informal caregivers of persons with dementia that can be used with caregivers from a variety of ethnicities.

**Methods:**

91 items were field tested by telephone interviews with 179 English-speaking and 21 monolingual Spanish-speaking caregivers of persons with dementia. Repeat interviews were conducted with 71 caregivers. Administration time, scale score distributions, item-scale correlations, reliability, and associations of scales with patient and caregiver demographic and caregiving characteristics were estimated. Structure of associations among scales was examined using exploratory factor analysis.

**Results:**

Item analysis yielded 80 items distributed across 10 scales, with median administration time of 17 minutes [IQR 13.5–22 minutes] and minimal missing data. There were few floor or ceiling effects in scale score distributions. Internal consistency reliability was ≥ 0.78 for all scales; test-retest reliability (intraclass correlation) estimates exceeded 0.70 for 6 scales. More hours weekly spent in caregiving was uniquely associated with worse quality of life on 8 scales (p's ≤ 0.05). Three higher-order dimensions of caregiving assistance, emotional and social concerns, and spirituality and benefits were identified.

**Conclusion:**

These preliminary results support subsequent evaluation of test-retest reliability, construct validity, and responsiveness to change of this quality-of-life measure for caregivers from diverse ethnicities.

## Background

The occurrence of Alzheimer's disease (AD) and related dementias accompanied by pervasive memory loss and associated behavioral disturbance is a major public health concern among older adults. The most rapidly growing segment of our population is that over the age of 80, and dementia is the most common cause of disability among these individuals [1].

Those with dementia eventually become totally dependent on one or more caregivers over the course of the illness. While a large proportion of persons with dementia are cared for in institutions, more than half receive care at home from spouses and other family members [2]. The largest proportion of patients with dementia receiving care in the home is among ethnic minorities, which significantly exacerbates challenged family dynamics via increased role and economic strains.

Providing care for individuals with a progressive, debilitating condition such as dementia can adversely impact the quality of life (QOL) of informal caregivers in many ways. Studies to assess the impact of new treatments or new ways of delivering care for people with dementia should incorporate instruments that very broadly address not only domains of health-related quality of life, but also non health-related quality of life, because these dimensions (for example, perceptions of family involvement in caregiving, caregiver's perceptions of having personal time away from caregiving) likely affect outcomes relevant to patients such as time to institutionalization. Our first step was to assess what kinds of caregiver measures were available in the literature. We conducted a literature search (updated in October 2008) using the PubMed, PsychINFO and AgeLine databases and using search terms *dementia caregiver *and *quality of life*, and we searched the MAPI Institute's online catalog of QOL measures . We found that with one exception, published studies of dementia caregiver quality of life had used generic quality-of-life measures or targeted narrower constructs of burden or depression. The only exception is a measure developed by the PIXEL study group [3].

Caregivers are more likely to be depressed and anxious and experience increased risk of physical health problems than non-caregivers [4]. Numerous research studies have demonstrated an association between caring for a loved one with dementia and the development of caregiver stress and burden [5]. Families often report that behavioral disturbances are the primary source of stress and the primary cause for institutionalization of their loved one [4,6,7]. Additionally, dementia has the effect of segregating caregivers from others because of the care needs and problem behaviors of dementia sufferers. Relationship strain between the caregiver and the dementia sufferer, and social isolation are negative consequences associated with assisting the dementia sufferer [8]. Furthermore, if the person with dementia is institutionalized, the caregiver may grieve for the loss of the relationship, another source of caregiver stress [9]. In effect, caregivers may experience declines in their physical, mental, and social health. These adverse health outcomes for caregivers may impact the use of patient services (including long-term care facilities) and outcomes of the person with dementia [6].

The construct of caregiver burden embodies only negative physical, psychological, social and financial demands of providing care for someone with dementia [5], for example, as assessed by the Zarit Caregiver Burden Interview [10]. Another negative caregiving outcome is depressive symptomatology, assessed using tools such as the Center for Epidemiologic Studies-Depression (CES-D) scale [11], the Geriatric Depression Scale (GDS) [12], or the Zung Depression Rating Scale (ZDRS) [13].

Measures of QOL include constructs such as physical, emotional and cognitive functioning, self-rated health, self-efficacy, spirituality, financial status, social support, and satisfaction with life situations among others [14,15]. Generic health-related quality of life measures used in studies of dementia caregivers include the EuroQol (EQ-5D) [16], Health Utilities Index Mark 2 (HUI-2) [17], and Short Form-36 Health Survey (SF-36) [18]. The sole published dementia-caregiving-targeted QOL measure was developed in the PIXEL study in France [3]. This instrument includes 20 dichotomous (yes/no) items that focus on four domains that are all negative aspects of caregiving (behavioral competence, relation to environment, psychological perceptions of the situation, and perception of distress) without addressing positive aspects such as benefits or spirituality and faith. The PIXEL measure does not capture the extent of care provided by the caregiver to the patient or assistance received by the caregiver either from family or another source.

We embarked on development of a comprehensive measure of both health-related and non-health-related quality of life for dementia caregivers, to fill the gap in the literature on such a tool. Our goal was to develop a measure that could be used in a diverse group of caregivers of multiple ethnicities and a wide range of education levels. We previously conducted six focus groups (2 groups predominantly Caucasian; 1 group Hispanic and conducted in Spanish; 1 group Chinese-American; 2 groups African-American) and 29 cognitive interviews (21 in English and 8 in Spanish) with ethnically diverse samples of caregivers of persons with a diagnosis of dementia. We then developed a draft set of caregiver quality-of-life items based on domains that were identified as key in assessing caregiver quality of life: caregiving assistance, personal time, family involvement, caregiving demands, worry, spirituality and faith, benefits of caregiving, caregiver feelings, and role limitations due to caregiving [19]. Spirituality and faith, and benefits of caregiving were domains uniquely identified by caregivers from minority groups. The goal of the study reported herein was to field test this item set, with the goal of developing a standardized caregiver quality of life instrument (CGQOL) in English and Spanish for use by the larger research community to assess quality of life for caregivers of persons with cognitive impairment.

## Methods

### Sample

Initially, 51 caregivers were recruited from consecutive patient/caregiver dyads enrolled in the UCLA Alzheimer's Disease Center longitudinal registry study [20]. The final 149 caregivers of persons with dementia or Alzheimer's disease were recruited using flyers posted in public locations and announcements in newsletters and websites. Criteria for enrollment required caregivers to be non-professional caregivers, be 18 years of age or older, live in Southern California, and be English or Spanish speaking. Informed consent was obtained from each subject prior to being interviewed at all time periods.

### Measures

A field test set of 91 items assessing aspects of caregiver quality of life was generated based on analysis of data from focus groups of caregivers from diverse ethnic groups in the southern California area [19]. These items tapped caregiving assistance (activities of daily living (ADLs) and instrumental activities of daily living (IADLs)), personal time, family involvement, caregiving demands, worry, spirituality and faith, benefits of caregiving, caregiver feelings, and role limitations due to caregiving. Some of the items and response scales were adapted from measures developed in the Medical Outcomes Study [21]. Field test items were translated into Spanish by one experienced translator, reviewed by a second translator, then administered to eight Spanish-speaking caregivers in a series of cognitive interviews, to assess and refine the wording [22,23]. Additional data that were collected by interview or from an existing database were patient and caregiver age, gender, ethnicity, marital status, and education; relationship of the caregiver to the patient; hours spent weekly in caregiving; duration of caregiving; average number of months as primary caregiver in the prior year; and unmet need for caregiving assistance. Unmet need was assessed by asking caregivers to rate the degree to which they were bothered by needing more help than was received and by the lack of help received from family members. Responses were provided on a 1–5 scale (not at all bothered = 1; extremely bothered = 5). An overall rating of the caregiving experience on a 0–10 scale (worst = 0; best = 10) and a rating of the severity of the patient's dementia (mild, intermediate, advanced) were also obtained.

### Data collection

An initial telephone interview was completed between May 2001 and July 2006. We collected these data by telephone interview because our goal was for the measure to be used in socioeconomically disadvantaged populations, including those with very low or no formal education and unable to read or write. This interview included demographic questions about the patient and caregiver, and the 91-question field test item set about quality of life as a caregiver for a person with dementia. The demographic questions in the interviews of the later group of 149 caregivers were expanded to include more questions about caregiver characteristics (age, marital status, ethnicity, education) than were available for the initial 51 subjects. Selected patient variables (age, marital status, ethnicity, and education) for the initial set of 51 caregivers were obtained from the Alzheimer's Disease registry database rather than from interview. A $10 payment was sent to each participant following each interview. To assess test-retest reliability, a subsample of 71 caregivers (51 English speaking and 20 Spanish speaking) completed a second telephone interview, identical to the first, 11 to 63 days after the initial interview (75% within 21 days).

Study activities were performed with the approval of the UCLA Office for Protection of Research Subjects (approval #G04-03-089-12).

### Analysis

Analyses were conducted using SAS version 9.1. All items were transformed linearly to have a 0–100 possible range, where higher values mean better caregiver quality of life in that domain [24]. Multitrait scaling was used to assess item convergence and discrimination across the ten dimensions/hypothesized scales [25]. Items that had higher correlations with a scale other than the hypothesized scale were moved, and the multitrait scaling analysis was run again. Items loading on more than one scale (applying in general a rule of loadings that were within one standard error or 0.076 of each other), and items that had no item-scale correlation greater than 0.35 were excluded from the final measure.

Scale scores were calculated by taking the mean of the final set of items included in each scale. For each scale the mean score, standard deviation (SD), observed minimum, and percent of subjects with minimum and maximum scores were calculated. Cronbach's alpha was used to estimate internal consistency for each multi-item scale [26]. Test-retest reliabilities and 95% confidence intervals around those estimates were generated using intraclass correlations between baseline and follow-up scores of 38 subjects who had a repeat interview within 21 days of the initial interview and who had a 1-point or no change in their overall rating of their caregiving experience (on a 0–10 scale) between the two interview dates.

Associations of patient and caregiver characteristics with caregiver quality-of-life scale scores were analyzed using Pearson or Spearman correlations or analysis of variance (ANOVA), as appropriate. Backwards stepwise regression was used to quantify the extent of unique relationships between each of the 10 caregiver quality-of-life scale scores (dependent variables) and these caregiver and patient characteristics: patient and caregiver age, gender, ethnicity, marital status, and education, caregiver's relationship to person with dementia, hours each week spent caring for person with dementia, number of years being a caregiver for person with dementia, and level of unmet need for caregiving assistance. For our bivariate and multivariate analyses of construct validity, we hypothesized that we would consistently observe associations of better quality-of-life with shorter duration of being a caregiver, fewer hours per week in caregiving, being a male caregiver, and milder dementia. Based on data from our earlier focus groups, we hypothesized that caregivers who were non-white would more strongly endorse spirituality and faith and benefits of caregiving compared to caregivers who were white. We anticipated that associations of caregiver quality of life with other caregiver and patient characteristics would be minimal or non-significant. Caregiver age, marital status, ethnicity and education were not collected for the initial group 51 of caregivers; patient age, gender, ethnicity, education and marital status were missing for a few patients in that caregiver subgroup, as well. Our imputation rule for subjects with missing data on these variables was to use the sample mean of subjects with non-missing data for the particular variable.

Higher order associations of individual scales were evaluated using factor analysis. Several number of factor criteria were run, including Guttman's weakest lower bound, Cattell's scree test, and parallel analysis. These indicated that 2- or 3-factor solutions were appropriate. Factor analysis was conducted using a Promax rotated factor solution.

## Results

Mean caregiver age was 61.5 years and mean patient age was 80.2 years (See Tables 1 and 2). Seventy-nine percent of caregivers and 57% of patients were female. The caregiver was a spouse of the patient 45% of the time, and 43% were an adult-child/child-in-law. Spanish was the primary language among 11% of the caregivers. Fifty-three percent of caregivers and 37% of patients had at least a 4-year college degree. Over half of caregivers provided more than 30 hours of caregiving each week, and 42% had been caregiving for more than 5 years. Of the 144 caregivers from whom the patient's dementia severity was asked in the interview, 17% indicated it was mild, 60% indicated it was intermediate, and 23% reported the severity of dementia as advanced.

**Table 1:** Caregiver Characteristics and Perceptions^a^

	**N (%)**
Age (mean, SD) *(n = 147)*	61.5 (13.5)

Gender *(n = 200)*	

Male	43 (22)

Female	157 (79)

Ethnicity *(n = 148)*	

White	97 (66)

African American	13 (9)

Asian	10 (7)

Hispanic	27 (18)

Other	1 (1)

Marital Status *(n = 178)*	

Married	115 (65)

Never Married	34 (19)

Separated	6 (3)

Divorced	18 (10)

Widowed	5 (3)

Education *(n = 147)*	

8th grade of less	5 (3)

Some high school	4 (3)

High school grad	12 (8)

Some college	48 (33)

4-year degree	43 (29)

More than 4-year degree	35 (24)

Relationship *(n = 200)*	

Spouse	89 (45)

Child/Child-in-law	85 (43)

Sibling/Sibling-in-law	7 (4)

Niece/Nephew	2 (1)

Grandchild	3 (2)

Friend	8 (4)

Other	6 (3)

Hours spent each week caring for relative/friend with dementia *(n = 200)*	

0 – 5 hours	18 (9)

6 – 10 hours	23 (12)

11 – 20 hours	26 (13)

21 – 30 hours	19 (10)

More than 30 hours	114 (57)

Time being a caregiver to relative/friend with dementia *(n = 200)*	

Less than a year	21 (11)

1 – 2 years	27 (14)

2 – 3 years	28 (14)

3 – 5 years	41 (21)

More than 5 years	83 (42)

Average number of months as primary caregiver during past year (mean, SD) *(n = 200)*	11.1 (3)

How much of the help you need in caring for your friend/relative with dementia do you receive? *(n = 200)*	

None	19 (10)

A little	50 (25)

Some	52 (26)

Quite a bit	38 (19)

As much as I need	41 (21)

Rating of caregiver experience (0 = worst experience possible, 10 = best experience possible) *(n = 199)*	

0	6 (3)

1	1 (1)

2	4 (2)

3	5 (3)

4	15 (8)

5	45 (23)

6	20 (10)

7	22 (11)

8	40 (20)

9	22 (11)

10	19 (10)

^a ^Caregiver age, gender, and ethnicity were only collected during the telephone interview of the later group of 149 caregiver enrollees. All other characteristics were obtained from all subjects during the telephone interview.

**Table 2:** Patient characteristics and perceptions ^a^

	**N (%)**
**Patient Characteristics**	

Age (mean, SD) *(n = 198)*	80.2 (10)

Gender *(n = 198)*	

Male	86 (43)

Female	112 (57)

Ethnicity *(n = 196)*	

White	140 (71)

African American	14 (7)

Asian	14 (7)

Hispanic	27 (14)

Other	1 (1)

Marital Status *(n = 198)*	

Married	104 (53)

Never Married	10 (5)

Separated	1 (1)

Divorced	18 (9)

Widowed	65 (33)

Education *(n = 194)*	

8th grade of less	17 (9)

Some high school	8 (4)

High school grad	58 (30)

Some college	40 (21)

4-year degree	41 (21)

More than 4-year degree	30 (16)

Dementia Severity *(n = 144)*	

Mild	25 (17)

Intermediate	86 (60)

Advanced	33 (23)

^a ^Dementia severity was only collected during the telephone interview of the later group of 149 caregiver enrollees. Patient age, gender, ethnicity, martial status and education were obtained from the Alzheimer's Disease registry database for the initial set of 51 caregivers (when available) and during the telephone interview for the later group of 149 caregivers.

There was only one missing or refusal response for the 91 items across the 200 subjects. Administration time was recorded for 76 subjects, and the median administration time for the 91 quality-of-life items and 19 other questions was 23.5 minutes (interquartile range 18.5 to 30).

We performed 8 iterations of multitrait scaling. Item analyses resulted in 80 final items (out of 91 in the field test item set) in the caregiver quality of life measure, distributed across 10 scales (See Table 3 and Additional Files 1 and 2: CGQOL 80-item Measure and CGQOL Scoring Manual): assistance with ADLs (5 items), assistance with IADLs (13 items), personal time (4 items), role limitations due to caregiving (5 times), family involvement (6 items), demands of caregiving (7 items), worry (9 items), caregiver feelings (20 items), spirituality and faith (3 items) and benefits of caregiving (8 items). Seven items were excluded because two item-scale correlations had similar loadings, and another four items were excluded because no item-scale correlation was greater than 0.35.

**Table 3:** Descriptive statistics and reliability estimates ^a^

Scale	No. of items	Mean (SD)	Observed minimum	% Scoring at floor (0 points)	% Scoring at ceiling (100 points)	Cronbach's alpha	Test-retest reliability (ICC, 95% CI) ^b^
Assistance in IADLS ^c^	13	21.6 (21.7)	0	20.0	0.5	0.88	0.86 (0.73 – 0.99)

Assistance in ADLS ^d^	5	63.0 (36.2)	0	12.5	26.5	0.93	0.86 (0.71 – 1.00)

Personal Time	4	45.2 (23.2)	0	2.0	0.0	0.78	0.63 (0.34 – 0.92)

Role Limitations Due to Caregiving	5	50.7 (24.2)	0	1.0	1.5	0.83	0.53 (0.20 – 0.86)

Family Involvement	6	52.6 (26.7)	0	1.0	6.5	0.86	0.74 (0.53 – 0.96)

Demands of Caregiving	7	50.9 (23.2)	3.6	0.0	2.0	0.86	0.72 (0.50 – 0.95)

Worry	9	50.2 (20.3)	2.8	0.0	0.0	0.82	0.53 (0.17 – 0.89)

Caregiver Feelings	20	66.0 (20.5)	1.3	0.0	1.0	0.94	0.65 (0.33 – 0.97)

Spirituality and Faith	3	63.5 (34.8)	0	12.1	26.1	0.92	0.83 (0.65 – 1.00)

Benefits of Caregiving	8	68.9 (23.0)	3.1	0.0	5.5	0.89	0.89 (0.79 – 0.99)

^a ^Higher values mean better functioning and well-being or **need for less assistance from caregiver**. n = 199 – 200 caregivers except for test-retest reliability

^b ^n = 38 caregivers with one point or less change in caregiver experience and 21 days or less (mean = 14.9 days, SD = 3.2) between baseline and retest.

^c ^IADLS = Instrumental Activities of Daily Living

^d ^ADLS = Activities of Daily Living

Mean scale scores ranged from 21.6 (assistance with IADLs) to 68.9 (benefits of caregiving). There were few floor and ceiling effects, with the largest ceiling effects on assistance with ADLs (27%) and on spirituality and faith (26%); the largest floor effect was on assistance with IADLs (20%). Cronbach's alpha ranged from 0.78 to 0.94; intraclass correlation coefficients for test-retest reliability ranged from 0.53 (role limitations due to caregiving; worry) to 0.89 (benefits of caregiving), exceeding 0.70 for 6 of the 10 scales (See Table 3).

A three-factor solution was interpreted as showing higher order dimensions of tangible assistance, psychosocial, and benefits/faith (See Table 4). Associations between factors ranged from 0.04 to 0.52.

**Table 4:** Promax rotated three factor solution for the 10 caregiver quality of life scales ^a^

**Scale**	**Tangible Assistance**	**Psychosocial**	**Benefits/Faith**
Assistance in IADLS ^b^	0.73		

Assistance in ADLS ^c^	0.47		

Personal Time	0.54		

Role Limitations Due to Caregiving	0.41	0.41	

Family Involvement		0.56	

Demands of Caregiving		0.75	

Worry		0.74	

Caregiver Feelings		0.77	

Spirituality and Faith			0.64

Benefits of Caregiving			0.66

^a ^Standardized regression coefficients of the factors on the scales. Factor pattern loadings > = 0.30 are shown.

Estimated correlations among factors: tangible assistance with psychosocial = 0.52, tangible assistance with benefits/faith = 0.04, psychosocial with benefits/faith = 0.18.

^b ^IADLS = Instrumental Activities of Daily Living

^c ^ADLS = Activities of Daily Living

Bivariate associations (See Additional File 3: Table S1) were most consistently observed between caregiver quality-of-life scales and hours per week spent caregiving, where more hours per week caregiving was negatively associated with all scales (p's < 0.01) except for spirituality and faith and for benefits of caregiving (p ≥ 0.05)). In contrast, there were few associations of duration of being a caregiver and caregiver quality-of-life scores. Non-white ethnicity of both the caregiver and of the patient were substantially associated with spirituality and faith and with benefits of caregiving (p's < 0.0001). Older caregivers had less worry, better family involvement, and provided less assistance with ADLs, but younger caregiving age was associated with higher spirituality and faith and higher benefits of caregiving scores (p's < 0.01). Less unmet need with caregiving assistance was associated with higher caregiver quality of life on 8 of 10 scales (all p ≤ 0.01).

Regression models confirmed the unique and consistent associations of more weekly caregiving hours with worse caregiver quality of life, except for no association with spirituality and faith or with benefits of caregiving (See Table 5). More unmet need for caregiving assistance was uniquely associated with 6 of the 10 scales (all p's < 0.05): personal time, role limitations due to caregiving, family involvement, demands of caregiving, caregiver worry, and caregiver feelings (See Table 5). Less patient education and non-white caregiver ethnicity were associated with higher spirituality and faith and higher benefits of caregiving scale scores. Demands of caregiving and caregiver feelings were worse for caregivers who were children.

**Table 5:** Caregiver quality of life scales regressed on patient and caregiver characteristics ^a^

	Adjusted R^2^	Unstandard-ized beta	Standard Error	t-test statistic	*P*-value
**Assistance in IADLS**	0.47				
Caregiving hours per week		-10.13	0.82	-12.42	< 0.001

**Assistance in ADLS**	0.30				
Patient male		12.87	4.78	2.69	0.008
Caregiving hours per week		-9.77	1.64	-5.97	< 0.001
Caregiver white		37.91	9.77	3.88	< 0.001

**Personal Time**	0.41				
Less unmet need for caregiving assistance		0.22	0.04	5.36	< 0.001
Caregiving hours per week		-7.76	0.94	-8.26	< 0.001

**Role Limitations Due to Caregiving**	0.27				
Less unmet need for caregiving assistance		0.18	0.05	3.76	< 0.001
Caregiver is child		-11.61	3.28	-3.54	< 0.001
Caregiver white		-7.97	3.66	-2.17	0.03
Caregiving hours per week		-6.93	1.09	-6.35	< 0.001

**Family Involvement**	0.37				
Less unmet need for caregiving assistance		0.38	0.05	7.45	< 0.001
Patient married		9.17	4.09	2.24	0.03
Caregiver is child		-13.76	3.77	-3.65	< 0.001
Caregiving hours per week		-2.22	1.11	-1.99	0.05

**Demands of Caregiving**	0.19				
Less unmet need for caregiving assistance		0.16	0.05	3.26	0.001
Caregiver male		7.71	3.64	2.12	0.04
Caregiver is child		-13.91	3.42	-4.07	< 0.001
Caregiving hours per week		-3.72	1.09	-3.39	0.001

**Worry**	0.21				
Less unmet need for caregiving assistance		0.10	0.04	2.44	0.02
Caregiving hours per week		-2.72	0.96	-2.84	0.005
Caregiver male		7.18	3.20	2.25	0.03
Caregiver is child		-10.09	4.11	-2.46	0.02
Caregiver at least college degree		6.84	3.16	2.16	0.03
Caregiver age		0.35	0.13	2.63	0.009

**Caregiver Feelings**	0.24				
Less unmet need to caregiver assistance		0.10	0.04	2.30	0.02
Patient male		-10.75	3.11	-3.45	0.001
Caregiver is child		-11.68	3.08	-3.80	< 0.001
Caregiving hours per week		-3.11	0.96	-3.25	0.001

**Spirituality and Faith**	0.27				
Patient age		-0.80	0.22	-3.67	< 0.001
Patient at least college degree		-20.03	4.58	-4.37	< 0.001
Caregiver white		-18.86	5.34	-3.53	< 0.001
Caregiver male		-14.44	5.18	-2.79	0.006
Caregiver married		-12.78	4.92	-2.59	0.01

**Benefits of Caregiving**	0.16				
Caregiver white		-15.89	3.90	-4.07	< 0.001
Patient at least college education		-6.67	3.17	-2.10	0.04
Caregiver age		-0.31	0.14	-2.19	0.03

^a ^n = 200. Ba ckwards stepwise regression models with p > 0.2 for removal from model. Listed independent variables with p < 0.05. Independent variables available for inclusion in the model: patient and caregiver age, gender, ethnicity, marital status, and education, caregiver's relationship to person with dementia, hours each week spent caring for person with dementia, number of years being a caregiver for person with dementia, and unmet need for caregiving assistance. Means were imputed for caregiver age, marital status, ethnicity and education and patient age, gender, marital status, ethnicity and education where these data were unavailable or missing.

## Discussion

We previously identified 10 dimensions of quality of life as relevant to caregivers of persons with dementia, based on focus groups of Hispanic, Caucasian, African-American, and Chinese-American caregivers. These dimensions were not limited to health-related aspects of quality of life, but were intended to incorporate non-health related issues and positive aspects of caregiving. Based on these focus group data, we developed a set of items; in this field test, we found that telephone administration of both English and Spanish-language versions of this item set was highly feasible, with only one of 200 subjects refusing to answer one item out of 91. We estimate median administration time for the 80 items to be 17.1 minutes (interquartile range estimated as 13.5 to 22 minutes). Examination of item-scale correlations enabled us to reduce the number of items in the final CGQOL measure to 80. Final scale score distributions were reasonable, with few floor or ceiling effects. Internal consistency reliability was excellent; however, test-retest reliability was adequate for only 6 of 10 scales. Because our sample was relatively small and confidence intervals around these estimates wide, further evaluation in a larger sample should be conducted.

We were able to assess selected aspects of construct validity in this study. As hypothesized [27],  of 8 of 10 scales in the CGQOL measure with extent of caregiving, as measured by weekly hours in caregiving, were strong; the two scales not associated with weekly hours in caregiving were those tapping more intangible aspects of caregiving: spirituality and faith, and benefits. However, in contrast to our expectations, duration of caregiving was not uniquely associated with caregiver quality of life, possibly because the associations were measured only at one point in time; it is likely that change in caregiver quality of life is associated with longer duration of caregiving. More severe dementia was highly associated with greater caregiving assistance with ADLs, although we hypothesized that the associations of dementia severity would be broader, spanning more dimensions of caregiver quality of life.

Stronger endorsements of spirituality and faith and of benefits of caregiving was positively associated with being a non-white caregiver, as hypothesized, and corroborating data from our earlier focus groups of African American and of Hispanic caregivers, which guided us to include these caregiving quality-of-life dimensions in the measure. The findings also emphasize the importance of including dimensions that are relevant to a diverse spectrum of caregivers.

There are limitations of the study that are important to acknowledge. There were not enough Spanish-language surveys to assess language equivalence across English and Spanish respondents. We recruited a convenience sample of caregivers in one geographic region, and there is potential bias in our sampling frame that could affect the generalizability of results. The single item on dementia severity was based on carer self-report and while it has face validity, its reliability and validity are unknown. Only 6 of 10 test-retest reliability point estimates exceeded the cutoff typically recommended as meeting standards for group comparisons [28]. However, we note that confidence intervals around the estimates were wide, and while we attempted to identify a subset less likely to have actually had change in quality of life by setting a cap on the test-retest interval and restricting to those who changed minimally on an external criterion variable, true change may have attenuated these estimates.

Because this instrument is targeted for dementia caregivers, future research should include administration of a brief generic measure of quality of life, to assess whether the generic health measure provides complementary information to this measure that broadly assesses both non-health-related and health-related aspects of quality of life of dementia carers. Future research efforts should also focus on assessing test-retest reliability in a larger sample to obtain more precise estimates, identifying a subset of items that performs as well as this 80-item version, a more extensive analysis of construct validity, evaluation of a self-administered version, and assessment of responsiveness to change.

## Conclusion

These preliminary results, while promising, should be followed by subsequent evaluation of test-retest reliability, construct validity, and responsiveness to change of this quality-of-life measure that is grounded in concerns of caregivers from diverse ethnicities.

## Competing interests

The authors declare that they have no competing interests.

## Authors' contributions

BGV planned the study, supervised data analyses, and wrote the paper. RDH also contributed to planning of the study, supervised data analyses, and contributed to revision of the paper. MLM helped design and supervised the research protocol, and contributed to writing the paper. SDV helped design and carried out the statistical analyses, and wrote the paper. LJF and TS helped with study planning and contributed to revision of the paper. All authors read and approved the final manuscript.

## Supplementary Material

Additional file 1 **CGQOL 80-item Measure**. Final version of the 80-item Caregiver-Targeted Measure of Quality of Life for Dementia Caregivers (CGQOL). Click here for file

Additional file 2 **CGQOL Scoring Manual**. Scoring Manual for Caregiver-Targeted Measure of Quality of Life for Dementia Caregivers (CGQOL). Click here for file

Additional file 3 **Table S1. Associations between caregiver and patient characteristics and caregiver quality of life scores**. Associations between caregiver and patient characteristics and caregiver quality of life scores. Click here for file
