# Supplementary material for: Development and preliminary evaluation of a quality of life measure targeted at dementia caregivers
Source: Health Qual Life Outcomes. 2009 Jun 21;7:56. doi: 10.1186/1477-7525-7-56 (PMC2706224; doi:10.1186/1477-7525-7-56)
Supplement: Additional file 1 — CGQOL 80-item Measure. Final version of the 80-item Caregiver-Targeted Measure of Quality of Life for Dementia Caregivers (CGQOL). [file 1477-7525-7-56-S1.pdf]

CAREGIVER QUALITY OF LIFE (CGQOL)

UCLA
Alzheimer Disease Center

Barbara G. Vickrey, MD, MPH
UCLA Department of Neurology
C-241 RNRC; Box 951769
Los Angeles, CA 90095-1769
Phone (310) 206-7671
Fax (310) 794-7716

INSTRUCTIONS:

This survey includes questions about your health and your experiences as a caregiver for a person with Alzheimer's disease or a related dementia.

Please answer every question by circling the appropriate number (1, 2, 3, ...).

If you are unsure about how to answer a question, please give the best answer you can and write a comment or explanation in the margin.

The following items are about activities with which you might assist your relative with dementia during a typical day. For each activity, please indicate how much you assisted that person during the last 4 weeks.

	Assisted a lot	Assisted a little	Did not assist at all?
1. Running errands	1	2	3
2. Paying bills	1	2	3
3. Arranging transportation for, or taking to appointments	1	2	3
4. Remembering names and dates	1	2	3
5. Using household appliances	1	2	3
6. Cleaning up around his or her home or residence	1	2	3
7. Answering the telephone	1	2	3
8. Making medical decisions	1	2	3
9. Making financial decisions	1	2	3
10. Preparing meals	1	2	3
11. Taking medications	1	2	3
12. Finding items in the home or residence	1	2	3
13. Helping him or her with recreational or social activities	1	2	3
14. Feeding him or her meals	1	2	3
15. Dressing	1	2	3
16. Bathing	1	2	3
17. Fixing hair, shaving, brushing teeth, applying make up	1	2	3
18. Using the toilet	1	2	3

During the last 4 weeks, did you have any of the following problems with your work or other regular daily activities as a result of being a caregiver?

	All of the time	Most of the time	Some of the time	A little of the time	None of the time
19. Cut down on the amount of time you spent on work or other activities	1	2	3	4	5
20. Accomplished less than you would like	1	2	3	4	5
21. Were limited in the kind of or other activities	1	2	3	4	5

	All of the time	Most of the time	Some of the time	A little of the time	None of the time
22. Had difficulty performing the work or other activities (for example, it took extra effort)	1	2	3	4	5
23. How much of the time during the last 4 weeks did you put your own needs aside to take care of your relative with dementia?	1	2	3	4	5

During the last 4 weeks, how satisfied were you with the following?

	Very dissatisfied	Somewhat dissatisfied	Neither satisfied nor dissatisfied	Somewhat satisfied	Very satisfied
24. How satisfied were you with the amount of personal time you had away from your relative with dementia? (that is, visiting friends or relatives, tending to personal affairs, enjoying hobbies, going to work, etc.)	1	2	3	4	5
25. Overall, how satisfied are you with the support you receive in caring for your relative with dementia?	1	2	3	4	5
26. How satisfied are you with the amount of help you receive from family in caring for your relative with dementia?	1	2	3	4	5
27. How satisfied are you with the quality of help you receive from family in caring for your relative with dementia?	1	2	3	4	5

During the last 4 weeks...

	Always	Often	Sometimes	Rarely	Never
28. how often were you able to spend enough time away from your relative with dementia?	1	2	3	4	5
29. how often did you feel “trapped” caring for the person with dementia?	1	2	3	4	5

	Always	Often	Sometimes	Rarely	Never
30. how often did you wish you could escape from your caregiving duties?	1	2	3	4	5
31. how often did you worry that you might be unable to take care of your relative with dementia in the future?	1	2	3	4	5
32. how often were you unsure about how to care for your relative with dementia?	1	2	3	4	5
33. how often did you worry about your relative with dementia?	1	2	3	4	5
34. how often did you worry about your relative's dementia getting worse?	1	2	3	4	5

How much are the following statements true or false for you?

	Definitely true	Mostly true	Don't know	Mostly false	Definitely false
35. During the last 4 weeks, I had as much time for myself as I wanted.	1	2	3	4	5
36. During the last 4 weeks, I received all the help I needed from family members in taking care of my relative with dementia.	1	2	3	4	5
37. Besides me, other relatives should help out more with caregiving.	1	2	3	4	5
38. I feel like I have not choice about being a caregiver.	1	2	3	4	5
39. Caregiving interferes too much with my life.	1	2	3	4	5
40. Too much of my time is spent caring for my relative with dementia.	1	2	3	4	5
41. My relative is a burden on me.	1	2	3	4	5
42. During the last 4 weeks, I worried about the financial cost of caring for my relative in the future.	1	2	3	4	5
43. During the last 4 weeks, I worried that someone else would harm or take advantage of my relative with dementia.	1	2	3	4	5

	Definitely true	Mostly true	Don't know	Mostly false	Definitely false
44. During the last 4 weeks, I worried that my relative with dementia would do something unsafe or hurt himself or herself.	1	2	3	4	5
45. My faith in a higher power gives me strength and comfort when caregiving becomes difficult.	1	2	3	4	5
46. My spiritual beliefs help me to cope with the challenges of caregiving.	1	2	3	4	5
47. My faith has grown stronger because of what has happened to my relative with dementia.	1	2	3	4	5
48. I am grateful for the time that I am able to spend with my relative with dementia.	1	2	3	4	5
49. Being a caregiver has enriched my life.	1	2	3	4	5
50. Being a caregiver has made my life more meaningful.	1	2	3	4	5
51. Being a caregiver has made me thankful for the time that I spend with my relative with dementia.	1	2	3	4	5
52. Being a caregiver has made me appreciate my life.	1	2	3	4	5
53. Being a caregiver has brought me closer to my relative with dementia.	1	2	3	4	5
54. I enjoy caring for my relative with dementia.	1	2	3	4	5
55. My relative with dementia is a blessing to me.	1	2	3	4	5

How much of the time during the last 4 weeks…

	None of the time	A little of the time	Some of the time	Most of the time	All of the time
56. did you spend personal time away from your relative with dementia?	1	2	3	4	5
57. how much did you feel like you were "trapped"?	1	2	3	4	5
58. how much did you worry about the safety of your relative with dementia?	1	2	3	4	5
59. how much have you felt unsure about how to care for your relative with dementia?	1	2	3	4	5
60. have you felt overwhelmed?	1	2	3	4	5

	None of the time	A little of the time	Some of the time	Most of the time	All of the time
61. how often did you awaken during the night and have trouble falling asleep again?	1	2	3	4	5
62. how often did you get the amount of sleep you need?	1	2	3	4	5
63. how often did you have trouble staying awake during the day?	1	2	3	4	5

Some people are bothered as a result of caring for a person with dementia, while others are not. How much are you bothered by the following areas?

	Not at all bothered	Somewhat bothered	Moderately bothered	Very much bothered	Extremely bothered
64. depression?	1	2	3	4	5
65. fatigue?	1	2	3	4	5
66. anxiety?	1	2	3	4	5
67. hopelessness?	1	2	3	4	5
68. anger?	1	2	3	4	5
69. stress?	1	2	3	4	5
70. loneliness?	1	2	3	4	5
71. embarrassment?	1	2	3	4	5
72. fear?	1	2	3	4	5
73. sorrow?	1	2	3	4	5
74. regret?	1	2	3	4	5
75. despair?	1	2	3	4	5
76. confused?	1	2	3	4	5
77. frustrated?	1	2	3	4	5
78. overwhelmed?	1	2	3	4	5
79. During the last 4 weeks, to what extent were you bothered by needing more help than you received?	1	2	3	4	5
80. During the last 4 weeks, to what extent were you bothered by the lack of help you received from other family members?	1	2	3	4	5
