# Supplementary material for: Development and preliminary evaluation of a quality of life measure targeted at dementia caregivers
Source: Health Qual Life Outcomes. 2009 Jun 21;7:56. doi: 10.1186/1477-7525-7-56 (PMC2706224; doi:10.1186/1477-7525-7-56)
Supplement: Additional file 2 — CGQOL Scoring Manual. Scoring Manual for Caregiver-Targeted Measure of Quality of Life for Dementia Caregivers (CGQOL). [file 1477-7525-7-56-S2.pdf]

STEP 1: RECODING ITEMS

ITEM NUMBERS	CHANGE ORIGINAL RESPONSE CATEGORY *	TO RECODED VALUE OF:
1, 2, 3, 4, 5, 6, 7, 8, 9, 10, 11, 12, 13, 14, 15, 16, 17, 18	1 →	0
	2 →	50
	3 →	100
28, 35, 36, 45, 46, 47, 48, 49, 50, 51, 52, 53, 54, 55, 57, 58, 59, 60, 61, 63, 79, 80	1 →	100
	2 →	75
	3 →	50
	4 →	25
	5 →	0
19, 20, 21, 22, 23, 24, 25, 26, 27, 29, 30, 31, 32, 33, 34, 37, 38, 39, 40, 41, 42, 43, 44, 56, 62, 64, 65, 66, 67, 68, 69, 70, 71, 72, 73, 74, 75, 76, 77, 78	1 →	0
	2 →	25
	3 →	50
	4 →	75
	5 →	100

* Precoded response choices as printed in the questionnaire.

STEP 2: AVERAGING ITEMS TO FORM SCALES

SCALE	NUMBER OF ITEMS	AFTER RECODING PER STEP 1, AVERAGE THE FOLLOWING ITEMS
Assistance with instrumental activities of daily living	13	1, 2, 3, 4, 5, 6, 7, 8, 9, 10, 11, 12, 13
Assistance with activities of daily living	5	14, 15, 16, 17, 18
Role limitations due to caregiving	5	19, 20, 21, 22, 23
Personal time	4	24, 28, 35, 56
Family Interaction	6	25, 26, 27, 36, 37, 80
Demands of caregiving	7	29, 30, 38, 39, 40, 41, 57
Worry	9	31, 32, 33, 34, 42, 43, 44, 58, 59
Spirituality and faith	3	45, 46, 47
Benefits of caregiving	8	48, 49, 50, 51, 52, 53, 54, 55
Caregiver feelings	20	60, 61, 62, 63, 64, 65, 66, 67, 68, 69, 70, 71, 72, 73, 74, 75, 76, 77, 78, 79
