# Supplementary material for: Development and preliminary evaluation of a quality of life measure targeted at dementia caregivers
Source: Health Qual Life Outcomes. 2009 Jun 21;7:56. doi: 10.1186/1477-7525-7-56 (PMC2706224; doi:10.1186/1477-7525-7-56)
Supplement: Additional file 3 — Table S1. Associations between caregiver and patient characteristics and caregiver quality of life scores. Associations between caregiver and patient characteristics and caregiver quality of life scores. [file 1477-7525-7-56-S3.pdf]

Table S1: Associations between caregiver and patient characteristics and caregiver quality of life scale scores [a]

	Assistance in IADLS	Assistance in ADLS	Personal Time	Role Limitations Due to Caregiving	Family Involvement	Demands of Caregiving	Worry	Caregiver Feelings	Spirituality and Faith	Benefits of Caregiving
**Caregiver Characteristics and Perceptions**										
Age (N = 147)	-0.039<br>p = 0.63	**0.236**<br>**p = 0.004**	-0.014<br>p = 0.86	0.088<br>p = 0.29	**0.245**<br>**p = 0.003**	0.032<br>p = 0.70	**0.273**<br>**p = 0.001**	0.153<br>p = 0.06	**-0.218**<br>**p = 0.008**	**-0.299**<br>**p = 0.0002**
Gender (N = 200)<br>1 = Male<br>0 = Female	0.079<br>p = 0.26	0.064<br>p = 0.37	0.133<br>p = 0.06	0.011<br>p = 0.88	0.052<br>p = 0.47	**0.196**<br>**p = 0.005**	**0.205**<br>**p = 0.004**	**0.268**<br>**p = 0.0001**	**-0.204**<br>**p = 0.004**	-0.010<br>p = 0.88
Ethnicity (N = 148)<br>1 = white<br>0 = non-white	0.104<br>p = 0.21	**0.327**<br>**p <.0001**	0.007<br>p = 0.94	-0.084<br>p = 0.31	0.185<br>p = 0.02	0.028<br>p = 0.74	0.169<br>p = 0.04	-0.020<br>p = 0.81	**-0.385**<br>**p <.0001**	**-0.393**<br>**p <.0001**
Marital Status (N = 178)<br>1 = married<br>0 = not married	-0.113<br>p = 0.13	**0.191**<br>**p = 0.01**	0.005<br>p = 0.95	0.094<br>p = 0.21	**0.230**<br>**p = 0.002**	0.112<br>p = 0.14	0.174<br>p = 0.02	0.164<br>p = 0.03	**-0.248**<br>**p = 0.001**	-0.160<br>p = 0.03
Education (N = 147)<br>1 = At least college degree<br>0 = Less than college degree	0.051<br>p = 0.54	0.066<br>p = 0.43	0.084<br>p = 0.31	-0.026<br>p = 0.76	0.060<br>p = 0.47	0.059<br>p = 0.48	**0.213**<br>**p = 0.01**	0.088<br>p = 0.29	-0.168<br>p = 0.04	-0.099<br>p = 0.23
Relationship (N = 200)<br>S = Spouse<br>C = Child<br>O = Other<br><br>F = F-statistic for ANOVA	Mean scores from Anova<br>S = 18.8<br>C = 25.5<br>O = 18.5<br>F = 2.4<br>p = 0.09	Mean scores from Anova<br>S = 70.0<br>C = 56.5<br>O = 60.4<br>F = 3.2<br>p = 0.04	Mean scores from Anova<br>S = 41.4<br>C = 47.9<br>O = 49.0<br>F = 2.2<br>p = 0.12	Mean scores from Anova<br>S = 52.0<br>C = 47.1<br>O = 57.7<br>F = 2.2<br>p = 0.11	**Mean scores from Anova**<br>**S = 62.1**<br>**C = 43.0**<br>**O = 50.3**<br>**F = 13.0**<br>**p <0.0001**	**Mean scores from Anova**<br>**S = 55.3**<br>**C = 44.5**<br>**O = 56.5**<br>**F = 5.8**<br>**p = 0.004**	**Mean scores from Anova**<br>**S = 54.1**<br>**C = 45.2**<br>**O = 53.0**<br>**F = 4.7**<br>**p = 0.01**	Mean scores from **Anova**<br>**S = 68.7**<br>**C = 60.8**<br>**O = 73.8**<br>**F = 5.6**<br>**p = 0.004**	Mean scores from Anova<br>S = 57.1<br>C = 68.1<br>O = 70.5<br>F = 2.8<br>p = 0.06	**Mean scores from Anova**<br>**S = 63.7**<br>**C = 71.2**<br>**O = 79.2**<br>**F = 5.5**<br>**p = 0.005**
Hours spent each week caring for relative/ friend with dementia (N = 200)	**-0.679**<br>**p < 0.0001**	**-0.403**<br>**p < 0.0001**	**-0.562**<br>**p < 0.0001**	**-0.426**<br>**p < 0.0001**	**-0.200**<br>**p = 0.005**	**-0.263**<br>**p = 0.0002**	**-0.249**<br>**p = 0.0004**	**-0.278**<br>**p < 0.0001**	0.141<br>p = 0.05	0.092<br>p = 0.20
Time being a caregiver to relative/ friend with dementia (N = 200)	**-0.192**<br>**p = 0.007**	-0.163<br>p = 0.02	-0.076<br>p = 0.28	-0.083<br>p = 0.24	-0.116<br>p = 0.10	-0.088<br>p = 0.22	-0.124<br>p = 0.08	-0.171<br>p = 0.02	-0.010<br>p = 0.88	0.017<br>p = 0.81
Average number of months as primary caregiver during past year (N = 200)	**-0.202**<br>**p = 0.004**	0.078<br>p = 0.27	**-0.198**<br>**p = 0.005**	-0.161<br>p = 0.02	-0.103<br>p = 0.15	-0.121<br>p = 0.09	-0.116<br>p = 0.10	-0.154<br>p = 0.03	-0.018<br>p = 0.80	-0.052<br>p = 0.46

Table S1: Associations between caregiver and patient characteristics and caregiver quality of life scales [a] (cont.)

	Assistance in IADLS	Assistance in ADLS	Personal Time	Role Limitations Due to Caregiving	Family Involvement	Demands of Caregiving	Worry	Caregiver Feelings	Spirituality and Faith	Benefits of Caregiving
Amount of help caregiver needed in caring that was received (0 - 100) (N=200) [b]	**0.192 p = 0.007**	**0.174 p = 0.01**	**0.391 p<0.0001**	**0.308 p < 0.0001**	**0.495 p < 0.0001**	**0.277 p < 0.0001**	**0.225 p = 0.001**	**0.193 p = 0.005**	-0.139 p = 0.05	-0.067 p = 0.34
Overall experience as a caregiver (0 - 10) (N=200) [c]	0.074 p = 0.30	-0.116 p = 0.10	**0.219 p = 0.002**	**0.251 p = 0.0003**	0.060 p = 0.40	**0.453 p < 0.0001**	**0.188 p = 0.008**	**0.388 p < 0.0001**	**0.199 p = 0.005**	**0.556 p < 0.0001**
**Patient Characteristics**										
Age (N = 198)	0.011 p = 0.88	-0.069 p = 0.92	0.009 p = 0.90	-0.087 p = 0.23	-0.117 p = 0.10	-0.117 p = 0.10	-0.026 p = 0.71	-0.101 p = 0.16	**-0.177 p = 0.01**	-0.044 p = 0.54
Gender (N = 198)<br>1 = Male<br>0 = Female	-0.089 p = 0.21	0.137 p = 0.06	**-0.202 p = 0.004**	-0.055 p = 0.44	0.131 p = 0.07	-0.009 p = 0.90	-0.049 p = 0.50	**-0.207 p = 0.003**	-0.059 p = 0.40	-0.083 p = 0.24
Ethnicity (N = 196)<br>1 = white<br>0 = non-white	0.085 p = 0.23	**0.213 p = 0.003**	0.031 p = 0.67	-0.034 p = 0.64	0.159 p = 0.03	0.074 p = 0.30	0.171 p = 0.02	0.016 p = 0.82	**-0.338 p <0.0001**	**-0.339 p < 0.0001**
Marital Status (N = 198)<br>1 = married<br>0 = not married	-0.088 p = 0.22	0.113 p = 0.11	-0.089 p = 0.21	0.067 p = 0.35	**0.339 p < 0.0001**	0.135 p = 0.06	0.147 p = 0.04	0.109 p = 0.13	-0.144 p = 0.04	-0.173 p = 0.02
Education (N = 194)<br>1 = At least college degree<br>0 = Less than college Degree	0.046 p = 0.52	**0.189 p = 0.01**	0.084 p = 0.24	0.011 p = 0.88	**0.273 p < 0.0001**	**0.179 p = 0.01**	**0.249 p=0.0005**	0.096 p = 0.19	**-0.348 p < 0.0001**	**-0.223 p = 0.002**
Dementia Severity (N = 144)<br>1 = Mild<br>2 = Intermediate<br>3 = Advanced	**-0.246 p = 0.003**	**-0.378 p < 0.0001**	-0.103 p = 0.22	-0.035 p = 0.68	-0.053 p = 0.53	0.039 p = 0.64	0.039 p = 0.65	0.059 p = 0.50	0.181 p = 0.03	0.103 p = 0.22

[a] Higher values for caregiver quality-of-life scale scores mean better functioning and well-being. Bolded values are Pearson associations where $p \leq 0.01$.

[b] Higher values mean less unmet need for caregiving assistance

[c] Higher values mean better overall experience as a caregiver.
